# Describing Chronic Malaria Infections

**DOI:** 10.4269/ajtmh.19-0085b

**Published:** 2019-04

**Authors:** George Dennis Shanks

**Affiliations:** Australian Defence Force Malaria and Infectious Disease Institute; Enoggera, Australia; E-mail: dennis.shanks@defence.gov.au

Dear Sir,

How one thinks about a problem is more important than the specific name used.^1,2^

Immunity implies a binary outcome, whereas tolerance is more indicative of a spectrum of outcomes. The purpose of presenting the old epidemiology data from Africa and Panama was to remind readers that even before molecular methods of parasite detection, malaria was largely a chronic infection with a variety of outcomes. Even under the worst possible conditions, the case fatality rate of falciparum malaria was relatively low as described in my other article in the same issue of *Am J Trop Med Hyg*.^3^

The practical importance of how one thinks about malaria infections is focused on current efforts to eradicate malaria. Is malaria immunity a hard-won status easily lost when infections are eliminated or does chronic parasitemia create tolerance which maintains an on-going risk of malaria-associated mortality? How one understands such a question is critical to malaria eradication and the commitment of large resources toward this goal.^4^ We should not condemn a large proportion of humanity to live in malaria-endemic areas because of our limited understanding of malaria immunity.
